# Volume Status in Severe Malaria: No Evidence Provided for the Degree of Filling of the Intravascular Compartment

**DOI:** 10.1371/journal.pmed.0020027

**Published:** 2005-01-25

**Authors:** Kathryn Maitland, Charles Newton, Kevin Marsh, Mike Levin

**Affiliations:** Centre for Geographic Medicine Research, Kenya Medical Research InstituteKilifiKenya; Faculty of Medicine and the Wellcome Trust Centre for Clinical Tropical MedicineLondonUnited Kingdom

The study by Planche et al. [1] provides important new information addressing intracellular volume depletion in children with severe childhood malaria, but does not address the question of whether intravascular volume depletion (hypovolemic shock) is present. Using sophisticated methodology to determine total body water and extracellular water, they demonstrate a 6.7% deficit in total body water and an 11.7% deficit of intracellular water, providing an important indication of the volumes of fluid that may be required to optimize hydration. The data, however, do not address the degree of filling of the intravascular compartment, nor should they be used to answer the question about the state of tissue and organ perfusion. Indeed, we believe that these new data present no conflict with our previously reported findings. Using methods to study critical illness physiology that are widely employed within pediatric intensive care units for interpretation of circulatory status, we have demonstrated evidence for hypovolemia in 53 Kenyan children with severe malaria complicated by metabolic acidosis [2]. Our children were younger, had longer capillary refilling times (>3 s), lower central venous pressures (mean 2.9 cm H_2_O) and higher creatinines (>80 µmol/l): all features of compensated hypovolemic shock. Furthermore, hypotension (systolic BP < 80 mm Hg) was present in 44% of children with severe acidosis (base deficit >15). These findings also indicate important baseline differences in two cohorts of children studied. We agree that reconsideration of guidelines for acute fluid management is warranted, particularly when current recommendations await an adequate evidence base. Nevertheless, conflicting opinions on the question of volume status in children with severe malaria can be satisfactorily resolved only through prospective randomized trials that include both fluid resuscitation and control groups. While the design and conduct of such trials will involve considerable challenges, optimal fluid management will never be resolved on the basis of theoretical consideration alone.
